# Signature of chronic hepatitis B virus infection in nails and hair

**DOI:** 10.1186/s12879-022-07400-8

**Published:** 2022-05-04

**Authors:** Haruki Komatsu, Ayano Inui, Enkhtaivan Odmaa, Yoshinori Ito, Hiroki Hoshino, Shuichiro Umetsu, Tomoyuki Tsunoda, Tomoo Fujisawa

**Affiliations:** 1 Department of Pediatrics, Toho University, Sakura Medical Center, 564-1 Shimoshizu Sakura, Chiba, 285-8741 Japan; 2 Department of Pediatric Hepatology and Gastroenterology, Eastern Yokohama Hospital, Yokohama, Kanagawa Japan; 3 National Center for Communicable Disease, Ulaanbaatar, Mongolia; 4 Department of Pediatrics, Nagoya University Graduate School of Medicine, Nagoya, Aichi Japan

**Keywords:** Hepatitis B virus, Hepatitis delta virus, Nail, Hair, Chimeric mouse, HBV DNA, HDV RNA, Phylogenetic tree analysis, Antiviral treatment, Hepatitis B surface antigen

## Abstract

**Background:**

Hepatitis B virus (HBV) is detected in extrahepatic tissues of individuals with HBV infection. Whether nails and hair contain HBV has been unknown.

**Methods:**

We examined two patient groups: those with chronic HBV infection alone (n = 71), and those with both chronic HBV and hepatitis delta virus (HDV) infections (n = 15). HBV DNA in the patients’ fingernails and hair were measured by real-time PCR. Hepatitis B surface antigen (HBsAg) of fingernails was evaluated by an enzyme immunoassay. HDV RNA in fingernails was measured by real-time PCR. Immunochemical staining was performed on nails. We used chimeric mice with humanized livers to evaluate the infectivity of nails.

**Results:**

Of the 71 pairs of HBV-alone nail and hair samples, 70 (99%) nail and 60 (85%) hair samples were positive for β-actin DNA. Of those 70 nail samples, 65 (93%) were HBV DNA-positive. Of the 60 hair samples, 49 (82%) were HBV DNA-positive. The serum HBV DNA level of the nail HBV DNA-positive patients was significantly higher than that of the nail HBV DNA-negative patients (p < 0.001). The hair HBV DNA-positive patients’ serum HBV DNA level was significantly higher compared to the hair HBV DNA-negative patients (p < 0.001). The nail HBV DNA level was significantly higher than the hair HBV DNA level (p < 0.001). The nails and hair HBV DNA levels were correlated (r = 0.325, p < 0.05). A phylogenetic tree analysis of the complete genome sequence of HBV isolated from nails and hair identified the infection source. Of the 64 nail samples, 38 (59%) were HBsAg-positive. All 15 pairs of chronic HBV/HDV infection nail and hair samples were β-actin DNA-positive. However, nail HBV DNA was detected in two patients (13%). None of the 15 patients were positive for hair HBV DNA. Nail HDV RNA was detected in three patients (20%). Of the 15 patients, eight (53%) were nail HBsAg-positive. HBsAg and hepatitis delta (HD) antigen were detected in the nails by immunochemical staining. Chimeric mice were not infected with PBS containing HBsAg and HBV DNA elucidated from nails.

**Conclusions:**

Nails and hair were the reservoir of HBV DNA. Moreover, nails can contain HBsAg, HDV RNA, and HD antigen.

**Supplementary Information:**

The online version contains supplementary material available at 10.1186/s12879-022-07400-8.

## Background

Hepatitis B virus (HBV) infection is one of the most serious infectious diseases worldwide. Humans can be vertically and horizontally infected with HBV due to exposure to blood. Body fluids such as saliva, semen, and tears from HBV carriers are also proven to be infectious vehicles [1–3]. The entry of HBV into cells is regulated by the viral receptor (sodium taurocholate co-transporting polypeptide: NTCP, designated as solute carrier family 10A1, SLC10A1) which is expressed predominantly in hepatocytes [4, 5]. However, many extrahepatic tissues have been reported to be positive for hepatitis B surface antigen (HBsAg) or HBV DNA [6–13]. Those reports suggest that HBV can spread to almost all extrahepatic tissues in HBV carriers.

Nails and hair are useful and valuable materials for medical investigation [14–17]. However, there are few investigations of nails and hair in HBV infection [18, 19]. In 2000, Nishiyori et al. reported that HBV DNA from fingernails was detected by a PCR technique in all of 20 patients whose sera were positive for HBsAg [18]. In 2018, Koroglu et al. reported that HBV DNA was detected on 27% of nail scissors and in 50% of nail specimens from 63 serum HBV DNA-positive patients [19]. Moreover, HBV carriers could be co-infected or superinfected with hepatitis delta virus (HDV). HDV is a defective RNA virus which requires the surface antigen of HBV for viral assembly and replication. Because HDV has the same envelope protein as HBV, both viruses share the specific viral receptor (NTCP) in liver [5]. However, it has not been known whether HDV RNA can be detected in nails.

The aim of this study is to clarify whether fingernails and hair could be reservoirs of HBV and HDV in patients chronic HBV mono-infection and HBV/HDV dual infection.

## Methods

In this study, the levels of HBV DNA in fingernails and hair were measured. Moreover, the levels of HDV RNA in fingernails were measured. The levels of HBsAg in the nails were also measured. We performed a phylogenetic tree analysis for the full-length genomic sequences of HBV and HDV RNA isolated from the patients’ nails and hair. Immunohistochemical staining was performed to detect HBV and HDV protein in the nails. Finally, the infectivity of fingernails was evaluated in chimeric mice with humanized livers.

### Patients and materials

Between February 2016 and April 2018, chronic HBV carriers who were positive for serum hepatitis B surface antigen (HBsAg) on two occasions at least 6 months apart were recruited at Japan’s Yokohama Eastern Hospital and Toho Sakura Medical Center. We also recruited patients with chronic HBV/HDV dual infections who were positive for serum HBsAg and anti-HDV IgG antibodies on two occasions at least 6 months apart at the National Center for Communicable Disease in Mongolia. The exclusion criteria for study participants were co-infection with hepatitis C virus, or human immunodeficiency virus, co-existing inherited causes of liver disease and malignant disease. Eligibility criteria were based on the Asian-Pacific clinical practice guidelines on the management of hepatitis B guidelines [20]. Seventy-one patients with chronic HBV infection alone (22 males, 49 females) were enrolled in this study. The majority of the patients were female because we have been following up HBV carrier mothers as well as children infected with HBV. The patients’ median age was 18 years (range 1–56 years). Additionally, fifteen (7 males, 8 females) with chronic HBV/HDV infection, ranging in age from 31 to 63 years old (median 43 years), were also enrolled in this study.

As negative controls, 15 subjects (male/female = 7/8, median age: 18 years) who were negative for serum HBsAg, anti-HBs, and anti-HBc were selected by matching ages in this study. Samples of the patients’ serum, fingernail (using a stainless steel clipper) and hair were collected by picking up at our hospitals. In order to avoid contamination, all of the nail and hair samples were washed three times with 1 mL of phosphate-buffered saline (PBS) each time and dried before being used in assays. The study protocols were approved by the ethical committee of Toho University Sakura Medical Center (no. 2015-073) and Eastern Yokohama Hospital (no. 2015010). This study was performed in accordance with the ethical guidelines of the 1975 Declaration of Helsinki. Written informed consent was obtained from all patients or legal guardians prior to sample collection.

### HBV DNA, HBV RNA, and HDV RNA extraction

From each patient, 0.5–1.0 mg of fingernails (nail plate) in weight and five 1-cm pieces of hair in length were used for DNA/RNA extraction for the quantification of HBV DNA, HBV pregenomic RNA (pg RNA), and HDV RNA. Hair with root was chosen for the extraction. Nail and hair DNA was extracted using the DNA Extractor FM kit (Wako Pure Chemical Industries, Osaka, Japan). The DNA extracted from nail and hair samples was dissolved in 50 µL of TE (Tris + EDTA) buffer. For the quantification of time-dependent nail HBV DNA, nails were incubated in 5 mL of recombinant cell dissociation reagent (TrypLE Select Enzyme 10X; Thermo Fisher Scientific, Waltham, MA). Then, nail HBV DNA was extracted from 200 µL of the cell dissociation reagent using a QIAamp DNA Blood Mini kit (Qiagen, Hilden, Germany). Serum HBV DNA was also extracted from 200 µL of serum using the QIAamp DNA Blood Mini kit (Qiagen). Finally, the extracted nail HBV DNA from the cell dissociation reagent and serum HBV DNA was dissolved in 100 µL of elution buffer.

For the extraction of HBV pgRNA from nails, the nail samples were resolved in 200 µL of the resolution buffer using DNA Extractor FM kit. The buffer containing resolved nail was then applied to an RNA extraction kit (Direct-zol RNA Mini Prep, Zymo Research, Irvine, CA). The RNA extracted from nail was dissolved in 50 µL of elution buffer. For the extraction of HDV RNA from nails, the recombinant cell dissociation reagent (TrypLE Select Enzyme 10×) was used. A small piece of nail (1–2 mm × 3–4 mm) was put into the tube containing 200 µL of the cell dissociation reagent and incubated at 37 °C overnight. Of the 200 µL of dissociation agent, 140 µL was applied to the QIAamp Viral RNA Mini kit (Qiagen). The RNA extracted from the nail was dissolved in 60 µL of elution buffer. The extracted RNA was treated with DNase. Isolated RNA was reverse transcribed with the random sequence anchored HBV pgRNA specific primer. The sequence of the HBV pgRNA specific primer for reverse transcription was 5′-ATTCTCAGACCGTAGCACACGACACGGAAAGAAGTCAGAAGGCAA-3′, in which the random sequence ATTCTCAGACCGTAGCACACGACAC was anchored at the 5′ end of the HBV-specific sequence GGAAAGAAGTCAGAAGGCAA (nucleotide [nt] 1974–1955) [21–23]. HDV RNA was reverse transcribed with random hexamer. The nt position of the HBV sequence was based on the genotype C gene (GenBank accession no. AB033550).

### Quantification of HBV DNA, HBV pgRNA, HDV RNA, and covalently closed circular DNA

Serum HBV DNA was quantified using the COBAS TaqMan HBV DNA test ver. 2.0 (Roche Diagnostics, Tokyo). The quantification of HBV DNA from nail and hair samples was performed with an in-house real-time assay [24]. These methods were previously described [25, 26].

Moreover, HBV covalently closed circular DNA (cccDNA), which accumulates cell nuclei and acts a template for the transcription of HBV viral genes, was quantified by real-time PCR [27]. For the quantification of cccDNA, DNA samples were incubated with T5 exonuclease (New England Biolabs, Ipswich, MA) to digest genomic DNA and viral relaxed circle DNA [28]. The recombinant plasmid controls were used for the quantification of cccDNA (Integrated DNA Technologies). The lower detection limit of the cccDNA assay was < 1000 copies/mL. Serum HDV RNA was measured by Fluorion HDV QLP 1.0 (Iontek, Istanbul, Turkey) with a lower detection limit of < 3500 IU/mL. The in-house real-time PCR assay for the quantification of nail HBV pgRNA and HDV RNA was based on the reported method [21–23, 29]. As an internal control for the RNA extraction from nail, mRNA of GAPDH was also amplified by real-time PCR.

In brief, cDNA from HBV pgRNA was amplified with forward primer (HBV-PG-F) [5′-CACCTCTGCCTAATCATC-3′] (nt: 1826–1843), reverse primer (random sequence) [5′-ATTCTCAGACCGTAGCACACGACAC-3′], and TaqMan probe (HBV-FM) [5′-FAM-TCCAAGCTGTGCCTT-MGB-3′] (nt: 1871–1885) [21–23]. The primers and probe for the real-time PCR of nail HDV RNA were same as the reported method [29]. As an internal control for RNA extraction from nail, mRNA of GAPDH was also amplified by real-time PCR (forward primer, 5′-CCTCCCGCTTCGCTCTCT-3′, reverse primer: 5′-GCTGGCGACGCA AAAGA-3′, probe: 5′-FAM-CCTCCTGTTCGACAGTCAGCCGC-3′-TAMRA).

Transcripts for use as the positive control for the HBV pgRNA and HDV RNA PCR assays were generated using a synthetic gene. We designed a synthetic gene containing the T7 promoter, forward and reverse sequence (Integrated DNA Technologies). The following synthetic sequence was used to generate the positive control for the HBV specific real-time PCR assay: *TAATACGACTCACTATAGGG*tttttcacctctgcctaatcatctcatgttcatgtcctactgttcaagcctccaagctgtgccttgggtggctttggggcatggacattgacccgtataaagaatttggagcttctgtggagttactctcttttttgccttctgacttctttccgtgtcgtgtgctacggtctgagaat (the length of the synthetic sequence was 199 bp. The use of italics indicates the T7 promotor sequence. The underlining indicates the HBV forward and reverse random sequence) and the HDV specific real-time PCR assay: *TAATACGACTCACTATAGGG*tggctctcccttagccatccgagtggacgtgcgtcctccttcggatgcccaggtcggaccgcgaggaggtggagatgccatgccgacccgaagaggaaagaagga (the length of the synthetic sequence was 125 bp. The use of italics indicates the T7 promotor sequence. The underlining indicates the HDV forward and reverse primer sequence). Transcripts were generated using the CUGA in vitro Transcription Kit (Nippon Gene, Tokyo) and then purified.

The transcripts were measured by a spectrophotometer, and the transcript copy number was calculated. The estimated transcript copy numbers of HBV pgRNA and HDV RNA were 1.16 × 10^14^ copies/mL and 1.29 × 10^14^ copies/mL, respectively. The lower detection limit of the HBV pgRNA and HDV RNA assay was < 1000 copies/mL. The real-time PCR was performed in a 25-μL reaction mixture containing 12.5 μL Premix Ex Taq (TaKaRa-Bio) with 0.4 μM of primers, 0.2 μM pf probes, and 3 μL of extracted DNA or cDNA. The real-time PCR program consisted of an initial pre-cycle incubation at 95 °C for 30 s, followed by 40 cycles of 95 °C for 5 s and 60 °C for 30 s. The PCR was performed in an MX3000P QPCR System (Agilent Technologies, Tokyo), and the results were analyzed with MxPro software (ver. 4.10). All assays were carried out in triplicate with negative control samples.

### Quantification of HBsAg in fingernails

To elute HBV proteins from nails, we used the recombinant cell dissociation reagent (TrypLE Select Enzyme 10×). A small piece of nail (1–2 mm × 3–4 mm) was put into a tube containing 300 µL of the cell dissociation reagent and incubated at 37 °C for 5 days. The reagent was then applied to the quantification of HBsAg. The HBsAg of fingernails was measured by the high-sensitivity HBsAg chemiluminescent enzyme immunoassay Lumipulse HBsAg-HQ (Fujirebio, Tokyo, Japan). This assay measures the concentrations of HBsAg within the range 0.005–150 IU/mL. We did not measure HBeAg, anti-HBe, anti-HBc, or anti-HDV in nails or hair.

### HBV and HDV full-length genome sequencing and phylogenetic analysis

To confirm whether nails and hair are useful to identify the infectious source of HBV infection, we retrospectively analyzed the cases of patients with chronic HBV infection through mother-to-child transmission. Of the 72 patients, four children (nos. 18, 19, 23 and 41) were evaluated for the phylogenetic tree analysis of HBV complete genome sequence. We also analyzed serum samples from all four mothers and nail samples from two mothers (no. 18 and no. 41). Thus, a total of 18 samples (serum: n = 8, nail: n = 6, hair: n = 4) were used for the analysis of the complete genome sequence of HBV. Moreover, the two HDV complete genome sequences were determined using nail HDV RNA from two patients (nos. HDV-6 and HDV-14). The complete genome sequences of HBV and HDV were amplified by the overlapping fragments method [30]. Similar to the HBV sequencing, the HDV compete genome was divided into the six overlapping regions. A nested PCR was performed to amplify the six regions [31]. The nested PCR primers are shown in Additional file 2: Table S1. The nucleotide positions of the primer sequences were based on the HDV complete genome (GenBank accession no. KF660600).

The PCR was performed using a 50-μL reaction mixture containing 25 μL of AmpliTaq Gold 360 Master Mix (Thermo Fisher Scientific), 0.4 μM of each primer, and 5 μL of cDNA. The first round of PCR amplification was performed as follows: initial pre-cycle incubation 95 °C for 10 min, 40 cycles of denaturation at 95 °C for 30 s, annealing at 50 °C for 30 s, and extension at 72 °C for 30 s. Then, 2 μL of the first PCR reaction product was re-amplified with inner primers for 40 cycles under the same reaction conditions as in the first-round PCR. The purified PCR products were directly sequenced by cycle sequencing. The method of phylogenetic tree analysis was previously described [26]. The nucleotide sequence data reported in this paper appear in the DDBJ/EMBL/GenBank nucleotide sequence databases with the accession numbers LC279245-62 (HBV), and LC426721-2 (HDV).

### Indirect immunogold labeling electron microscopy

For electron microscopy (EM), a piece of fingernail was put into a tube containing 100 µL of recombinant cell dissociation reagent (TrypLE Select Enzyme 10×) and incubated at 37 °C for 24 h. To confirm the existence of HBV in the reagent, we performed indirect immunogold labeling EM as described [25]. A drop of the incubated cell dissociation reagent was placed on a carbon-coated 200-mesh nickel grid (JEOL, Tokyo). Primary antibody solution (human anti-HBsAg immunoglobulin, Nihon Pharmaceutical, Tokyo, diluted 1:30 in PBS) and secondary antibody solution (goat polyclonal secondary antibodies to human IgG, 6 nm Gold; Jackson ImmunoResearch, West Grove, PA; diluted 1:100 in PBS) were used for EM.

### Immunohistochemical staining for fingernail samples

Formalin-fixed paraffin-embedded sections of fingernail were deparaffinized with xylene. The primary antibody for the detection of HBsAg and hepatitis D (HD) antigen in the fingernail was mouse anti-HBsAg monoclonal antibody (sc-57785, Santa Cruz Biotechnology, Santa Cruz, CA) at the concentration of 2 µL/mL and mouse anti-HDV monoclonal antibody (MC406.3, Gentaur Molecular Products, Brussels, Belgium) at the concentration of 10 µL/mL, respectively. Mouse anti-HBcAg monoclonal antibody (ab8638, Abcam, Cambridge, UK) and rabbit anti-SLC10A1 polyclonal antibody (ab131084, Abcam) were used as the primary antibodies for the detection of HBcAg and NTCP, respectively. As a negative control for the primary antibodies, mouse IgG1 (X0931, Dako, Glostrup, Denmark) was used. Biotin-labeled goat anti-mouse IgG (BA-9200, Vector Laboratories) was used as the secondary antibody.

### Inoculation of chimeric mice and the real-time PCR for the mouse samples

Three female chimeric mice with humanized liver were purchased from Phoenix Bio (Hiroshima, Japan). The data of the three chimeric mice (Nos. 101, 201, and 202) on the 1st day of inoculation were as follows: body weight, 21.4, 21.7 and 21.5 g; serum human-albumin levels; 10.9, 12.0, and 11.7 mg/mL. Of the three mice, one (no. 101) was intravenously once inoculated with the negative control. The other two mice (Nos. 201 and 202) were intravenously inoculated once with supernatant of PBS incubated with fingernail. The measurement of serum HBV DNA was previously described [25]. These chimeric mice were kept in a clean room and supplied with sterilized laboratory chow and water. The mice were anesthetized with isoflurane and sacrificed. All animal experiments were performed in accordance with both the Guidelines for Animal Experimentation of the Japanese Association for Laboratory Animal Science and the recommendations in the Guide for the Care and Use of Laboratory Animals of the National Institutes of Health, and under the approval of the Ethics Review Committee for Animal Experimentation of Phoenix Bio (No.1853).

### Statistical analyses

Categorical variables were compared between groups using the Yates corrected chi-square test or Fisher’s exact test. Non-categorical variables were compared between groups by the Mann–Whitney U-test. For the correlations between two variables, we used Spearman rank-order correlation coefficient. All tests were two-sided, and p-values < 0.05 were considered significant. All statistical analyses were performed with StatMate IV for Windows (Advanced Technology for Medicine & Science, Tokyo) and Microsoft Office Excel 2007.

## Results

### Patients

Of the 71 patients with chronic HBV infection alone, 50 (70%) were positive for serum HBeAg. Their serum HBV DNA levels ranged from 2.3 to > 9.0 log copies/mL (median > 9.0 log copies/mL). Serum HBV DNA level was significantly higher in HBeAg- positive patients (median > 9.0 log copies/m) than HBeAb-positive patients (median 3.5 log copies/mL). Of the 71 patients, seven (10%) were classified as having HBV genotype B. The remaining 64 (90%) was classified as having HBV genotype C.

All of the 15 patients with chronic HBV/HDV infection were positive for anti-HBe antibodies and serum HBV DNA. Their serum HBV DNA levels ranged from 2.1 to 4.4 log copies/mL (median 2.6 log copies/mL). All but two of these patients were positive for serum HDV RNA; their serum HDV RNA levels ranged from 4.9 to 8.0 log IU/mL (median 5.9 log IU/mL). The HBV genotype was unknown in all 15 patients with chronic HBV and HDV dual infection.

### β-Actin DNA and HBV DNA in nails and hair from the chronic HBV mono-infection

Seventy-one pairs of fingernails and hair samples were obtained from the chronic HBV mono-infection patients. Of the 71 pairs of nails and hair, 70 (99%) and 60 (85%) were positive for nails and hair β-actin DNA, respectively. There was a significant difference in the positive rate of β-actin DNA between the nails and the hair (p < 0.05). We therefore measured HBV DNA in the 70 nail samples (HBeAg positive; n = 50) and 60 hair samples (HBeAg positive; n = 45) that were positive for β-actin DNA. As negative controls, moreover, 15 pairs of fingernails and hair samples were obtained from subjects who were negative for all serum HBV markers. Of the 15 pairs of nails and hair, 14 (93%) and 11 (73%) were positive for nails and hair β-actin DNA. The 15 nail samples and 11 hair samples that were positive for β-actin DNA were evaluated for HBV DNA.

Of the 70 nail samples, 65 (93%) were positive for HBV DNA, and of the 60 hair samples, 49 (82%) were positive for HBV DNA; this difference in the positive rate was not significant (p = 0.05). The median HBV DNA level was 5.0 log copies/mL (range 3.0–6.9 log copies/mL) in nails and 4.3 log copies/mL (range 2.3–5.8 log copies/mL) in hair, showing a significant difference in the HBV DNA level between nails and hair (p < 0.001). These findings suggest that nails could reserve greater amounts of HBV DNA compared to hair. Moreover, there was a significant difference in the positive rate of nail/hair HBV DNA between serum HBeAg-positive and -negative patients (Tables 1 and 2). All of the 14 nail and 11 hair samples from negative control subjects were negative for HBV DNA. Additionally, PBS used for washing nails and hairs was evaluated by HBV real time-PCR assay. Thirty-two collected PBS samples which were used for washing the HBV DNA-positive nail and hair samples (nail: n = 16, hair: n = 16) were evaluated. All of the PBS samples were negative for HBV DNA.

**Table 1:** Comparison of the positive rate of nail HBV DNA between serum HBeAg-positive and -negative patients

		HBeAg in serum	
		Positive	Negative
HBV DNA in nail	Positive	49	16
	Negative	1	4
			p < 0.05

**Table 2:** Comparison of the positive rate of hair HBV DNA between serum HBeAg-positive and -negative patients

		HBeAg in serum	
		Positive	Negative
HBV DNA in hair	Positive	40	9
	Negative	5	6
			p < 0.05

### Correlation of the HBV DNA levels among serum, nails, and hair in the chronic HBV mono-infection patients

The results of our comparison of the serum HBV DNA levels between nail/hair HBV DNA-positive and -negative patients are summarized in Table 3. A serum HBV DNA level > 9.0 log copies/mL was considered to be 9 log copies/mL. The median of the serum HBV DNA level (9.0 log copies/mL) in the 65 nail HBV DNA-positive patients was significantly higher than that (3.0 log copies/mL) in the five nail HBV DNA-negative patients (p < 0.001). Similarly, the median of the serum HBV DNA level (9.0 log copies/mL) in the 49 hair HBV DNA-positive patients was significantly higher than that (4.3 log copies/mL) in the 11 hair HBV DNA-negative patients (p < 0.001). Of the five patients who were negative for nail HBV DNA, four were also negative for hair HBV DNA. These findings suggest that the patients with a high viral load have a tendency to become positive for both nail and hair HBV DNA.

**Table 3:** Comparison of HBeAg in serum and serum HBV DNA level between nail/hair HBV DNA -positive and -negative patients

	PCR assay for nail HBV DNA		PCR assay for hair HBV DNA	
	Positive	Negative	Positive	Negative
	n = 65	n = 5	n = 49	n = 11
HBeAg positive in serum	49	1	40	5
Serum HBV DNA level*				
Median, log copies/mL	9	3	9	4.3
(range)	(2.6 to > 9.0)	(2.3 to 4.3)	(2.6 to > 9.0)	(2.3 to > 9.0)

p < 0.001 in both nail and hair

*Serum HBV DNA level > 9.0 log copies/mL is considered to be 9 log copies/mL

**Fig. 1:**
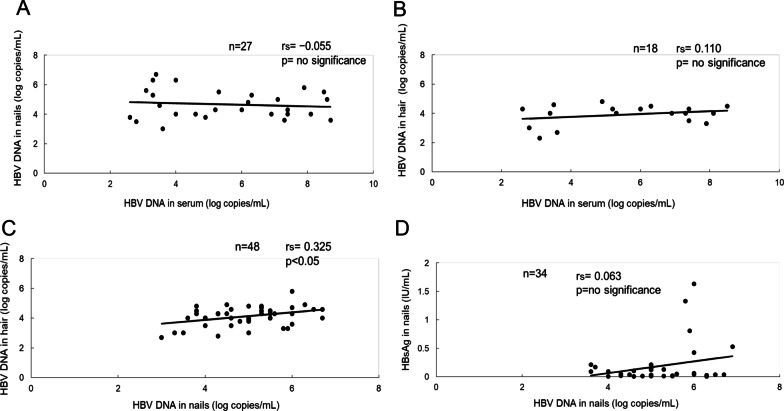
**A** The correlation between serum HBV DNA and nail HBV DNA. No significant correlation was observed (n = 27, r =  − 0.111, p = 0.582). **B** The correlation between serum HBV DNA and hair HBV DNA. No significant correlation was observed (n = 18, r = 0.285, p = 0.251). **C** The correlation between nail HBV DNA and hair HBV DNA. There was a weak but significant correlation between nail HBV DNA and hair HBV DNA (n = 48, r = 0.378, p < 0.01). **D** The correlation between nail HBV DNA and hair HBsAg. No significant correlation was observed (n = 34, r = 0.255, p = 0.145)

We also evaluated the correlation of the serum HBV DNA level with the nail HBV DNA level and hair HBV DNA level. The patients whose serum HBV DNA levels were > 9 log copies/mL were excluded from the evaluation. We thus evaluated the correlation between serum and nail HBV DNA and the correlation between serum and hair HBV DNA in 27 and 18 patients, respectively. The nail HBV DNA level showed no significant correlation with the serum HBV DNA level (Fig. 1A). Similarly, the hair HBV DNA level showed no significant correlation with the serum HBV DNA level (Fig. 1B). Although nail and hail HBV DNA were significantly detectable in the patients with high viral loads, neither the nail nor the hair HBV DNA level correlated significantly with the serum HBV DNA level. These findings suggest that the upper limitation of the HBV DNA level was present in the nails and hair.

The correlation between nail and hair HBV DNA level was evaluated. Of the 71 patients, 48 were positive for both nail and hair HBV DNA. The weak but significant correlation between the nail and hair HBV DNA levels is illustrated in Fig. 1C. (r = 0.325, p < 0.05). These findings indicate that the kinetics of HBV DNA in nails is closely related to the kinetics in hair. The correlation between the nail HBV DNA level and the nail HBsAg level is illustrated in Fig. 1D. There was no significant correlation between these levels. These findings indicate that the kinetics of nail HBV DNA are different from those of nail HBsAg.

### HBV cccDNA and HBV pregenomic RNA in nails/hair from chronic HBV mono-infection

The nail samples with an HBV DNA level ≥ 5 log copies/mL were evaluated for the detection of HBV cccDNA and HBV pgRNA. Twenty-two nail samples (nail HBV DNA levels: median 6 log copies/mL, range 5–6.9 log copies/mL) which were positive for mRNA of GAPDH were available for the real-time PCR for cccDNA. All but one was negative for cccDNA or HBV pgRNA. Only one sample, which showed the highest nail HBV DNA level (6.9 log copies/mL) in this study, was positive for both cccDNA and HBV pgRNA. The levels of cccDNA and HBV pgRNA in nail were 3.3 log copies/mL (Additional file 1: Fig. S1A) and 5.5 log copies/mL (Additional file 1: Fig. S1B–D), respectively.

We used the hair samples with an HBV DNA level ≥ 4.5 log copies/mL for the evaluation of HBV cccDNA. Eleven hair samples (hair HBV DNA levels: median 4.6 log copies/mL, range 4.5–4.9 log copies/mL) were available for the real-time PCR for cccDNA and HBV pgRNA. None of the 11 hair samples was positive for cccDNA. Because cccDNA was not detected in hair, a real-time PCR assay for HBV pgRNA in hair was not performed. These findings suggest that HBV could replicate in nails, but not in hair.

### Nails from the chronic HBV mono-infection group incubated with cell dissociation reagent

To evaluate the time-dependent changes of the HBV DNA, HBsAg, and β-actin DNA levels in the cell dissociation reagent mixed with nails, we place four nail samples (Patient A, B, C, and D, each sample: 0.5–1.0 mg of fingernail) into individual tubes each containing 5 ml of the cell dissociation reagent and incubated the tubes for 7 days (Table 4). PBS was used as a control. At each time point, 500 µL of the cell dissociation reagent was taken from the tube and used for assays: 300 µL for HBsAg, 200 µL for DNA extraction. At 0 h, all four of the samples were negative for HBV DNA and positive for β-actin DNA. With time, all four patients showed a rising trend in HBV DNA, HBsAg, and β-actin DNA level. In contrast to the cell dissociation reagent, all but one of the four samples incubated with PBS (Patient C) were negative for β-actin DNA at 0 h, and all were negative for HBV DNA and HBsAg at 0 h. Compared to PBS, the use of the cell dissociation agent could definitely elute HBV DNA and HBsAg from nails, with time. These findings suggest that HBV DNA and HBsAg are integrated into nail tissue. Interestingly, PBS was able to elute HBV DNA and HBsAg from nails; this indicates that nail tissue seems to be more fragile than we expected.

**Table 4:** HBV DNA and HBsAg level in nails mixed with cell dissociation reagent and PBS with time

	Incubation time	Patient A			Patient B			Patient C			Patient D		
		HBV DNA	HBsAg	β-actin	HBV DNA	HBsAg	β-actin	HBV DNA	HBsAg	β-actin	HBV DNA	HBsAg	β-actin
		log copies/mL	IU/mL	log copies/mL	log copies/mL	IU/mL	log copies/mL	log copies/mL	IU/mL	log copies/mL	log copies/mL	IU/mL	log copies/mL
Cell dissociation reagent	0 h	ND	ND	3.9	ND	ND	3.6	ND	ND	4	ND	0.023	3.3
	1 h	3.7	0.023	5.5	3.6	ND	4.8	3.6	0.031	5.8	2.9	0.047	5.5
	3 h	3.8	0.034	5.7	3.8	0.023	5	3.4	0.025	6	3.4	0.137	5.8
	6 h	4.1	0.032	5.8	3.9	0.052	5.3	3.1	0.024	6	3.6	0.296	5.8
	12 h	3.9	0.033	5.7	4.3	0.136	5.3	2.8	0.023	6	3.8	0.798	6
	24 h	4	0.028	5.7	4.6	0.402	5.7	3.1	0.025	6	4.5	1.16	6.3
	Day 3	4	0.03	5.8	5	0.864	6	3.2	0.025	6	4.5	1.56	6.5
	Day 5	3.9	0.045	5.7	5.1	1.12	6.3	3.3	0.022	6	4.5	1.54	6.6
	Day 7	3.8	0.048	5.7	5.2	1.32	6.3	3.2	0.021	6	4.5	1.33	6.6
PBS	0 h	ND	ND	ND	ND	ND	ND	ND	ND	5.7	ND	ND	ND
	1 h	3.3	ND	4.3	ND	ND	ND	ND	ND	5	ND	ND	4.3
	3 h	3.8	ND	4.8	ND	ND	ND	ND	ND	4.9	ND	ND	4.3
	6 h	3.3	ND	4	ND	ND	3.5	2.8	ND	4.7	ND	ND	4.3
	12 h	3.4	ND	4.6	ND	ND	3.8	ND	ND	4.6	ND	ND	4.7
	24 h	3.8	ND	4.6	ND	ND	3.7	ND	ND	4.5	ND	ND	4.5
	Day 3	4	ND	4.8	ND	ND	3.7	ND	ND	4.6	3	ND	4.9
	Day 5	4.1	ND	5	ND	ND	4.3	ND	ND	4.8	3.7	0.02	4.8
	Day 7	4.8	ND	6	ND	ND	4.8	3.6	ND	4.5	4.8	0.1	5.5

*ND* not detectable

### HBsAg levels in nails from the chronic HBV mono-infection patients

Seventy-one nail samples from the chronic HBV mono-infection patient were collected for this study. However, we used up 7 nail samples for the PCR analysis. Thus, the 64 nail samples were available for the measurement of HBsAg. Of the 71 nail samples from the chronic HBV mono-infection patients, 64 (HBeAg-positive, n = 46) were available for the measurement of HBsAg. Of those 64 nail samples, 38 (59%) (HBeAg-positive, n = 29) were positive for nail HBsAg. The levels of HBsAg ranged from 0.005 to 1.630 IU/mL (median 0.0465 IU/mL). The median serum HBV DNA level (9.0 log copies/mL) of the nail HBsAg-positive patients was higher than that (7.35 log copies/mL) of the nail HBsAg-negative patients, but not significant (p = 0.088). In addition, there was no significant difference in the positive rate of nail HBsAg between serum HBeAg-positive and -negative patients (p = 0.501).

The association between the detection of HBV DNA in nail/hair and the detection of HBsAg in nail is summarized in Table 5. There was no significant difference in the nail HBV DNA-positive rate between the nail HBsAg-positive and -negative samples. Moreover, there was no significant difference in the hair HBV DNA positive rate between the nail HBsAg-positive and -negative samples. These findings suggest that the presence of HBsAg in nail cannot predict the presence of HBV DNA in nail and hair.

**Table 5:** Association between the detection of HBV DNA in nail/hair and the detection of HBsAg in nails

HBV DNA in nail/hair	HBsAg in nails			
	Positive, n = 38		Negative, n = 26	
	Positive	Negative	Positive	Negative
Nails	34	4	25^‡^	0^‡^
Hair	26^†^	8^†^	19^§^	1^§^

The nail HBV DNA level of the 34 nail HBsAg-positive patients ranged from 3.6 to 6.9 log copies/mL (median 5.0 log copies/mL). The nail HBV DNA level of the 25 nail HBsAg-negative patients ranged from 3.0 to 6.7 log copies/mL (median 4.3 log copies/mL). There was a significant difference in the nail HBV DNA level between the nail HBsAg-positive and -negative patients (p < 0.01)

^†^Of the 38 patients, 4 were negative for hair β-actin PCR

^‡^Of the 26 patients, one was negative for nail β-actin PCR

^§^Of the 26 patients, 6 were negative for hair β-actin PCR

### Nails/hair HBV DNA and the nail HBsAg level during and after antiviral treatment

**Fig. 2:**
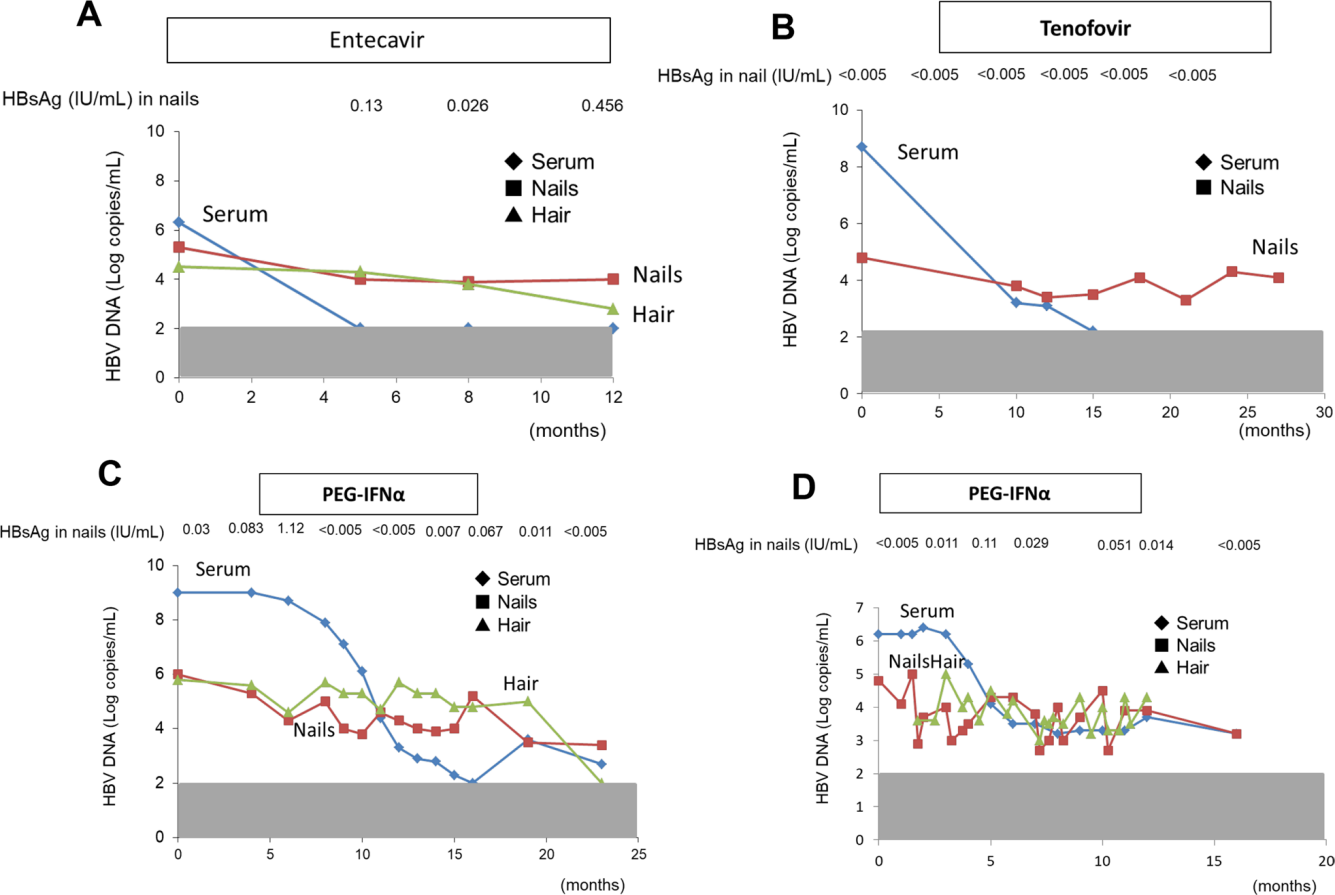
The changes of nail/hair HBV DNA and nail HBsAg level due to the antiviral treatment. Clinical courses of five patients are shown. **A** Male; 20 years old, **B** female; 24 years old, **C** female; 6 years old, **D** female; 20 years old

Of the 71 patients with chronic HBV mono-infection, four had received antiviral therapy between 2016 and 2017. Of the four patients who were treated with antiviral therapy, two received oral antiviral drugs (entecavir and tenofovir) and two received pegylated interferon-alfa (PEG-IFNα). The clinical courses of the four patients are summarized in Fig. 2. In the two patients treated with oral antiviral drugs, the nail HBV DNA remained positive for over several months after the disappearance of serum HBV DNA (Fig. 2A, B).

In contrast to the oral antiviral drugs, the two patients treated with PEG-IFNα did not become negative for serum HBV DNA during the treatment. However, their serum levels of HBV DNA fell significantly after the initiation of PEG-IFNα therapy (Fig. 2C, D). Despite the rapid reduction of their serum HBV DNA levels, their nail and hair HBV DNA levels showed no rapid reduction during or after the PEG-IFNα treatment. However, there was a significant association between hair/nail HBV DNA level and elapsed time (nails: n = 14, rs = − 0.654, p < 0.05, hair: n = 14, rs = − 0.552, p < 0.05) in Fig. 2C. In contrast, there was no significant association between hair/nail HBV DNA level and elapsed time (nails: n = 22, rs = − 0.273, hair: n = 22, rs = − 0.244) in Fig. 2D.

### HBV DNA, HDV RNA, and HBsAg in nails and HBV DNA in hair from the patients with chronic HBV/HDV dual infections

Fifteen pairs of fingernails and hair were obtained from the 15 patients with chronic HBV/HDV infection. β-actin DNA was detected in all of the nail and hair samples. However, nail HBV DNA and nail HDV RNA was detected in two (13%) and three (20%) of the 15 patients, respectively. There was only one patient (no. HDV-14) who was positive for both nail HBV DNA and nail HDV RNA. All of 15 nail samples from the negative control subjects were negative for HDV RNA. HBsAg was detected in the nails of eight (53%) of the 15 patients. The range of HBsAg in the nail samples ranged from 0.006 to 0.034 IU/mL (median 0.021 IU/mL). The characteristics of the nail HBV DNA- and nail HDV RNA-positive patients are listed in Table 6. All four (male/female = 3/1, age range 44–54 years) of the patients who were positive for nail HBV DNA and/or nail HDV RNA were positive for nail HBsAg. The levels of nail HBV DNA were 3.3 log copies/mL (HDV-11) and 4.0 log copies/mL (HDV-14). The levels of nail HDV RNA were 6.7 log copies/mL (HDV-6), 5.0 log copies/mL (HDV-13), and 6.7 log copies/mL (HDV-14). None of the 15 patients were positive for hair HBV DNA.

**Table 6:** Characteristics of nail HBV DNA positive and/or nail HDV RNA positive patients

Patient no.	Serum HBV DNA level	Nail HBsAg level	Nail HBV DNA level	Serum HDV RNA level	Nail HDV RNA level
	log copies/mL	IU/mL	log copies/mL	log IU/mL	log copies/mL
HDV-6	2.6	0.011	Neg	Neg	6.7
HDV-11	3	0.024	3.3	6	Neg
HDV-13	2.6	0.011	Neg	6.7	5
HDV-14	2.3	0.03	4	8	6.7

*Neg* negative

### Phylogenetic analysis of the HBV and HDV complete genome sequences

**Fig. 3:**
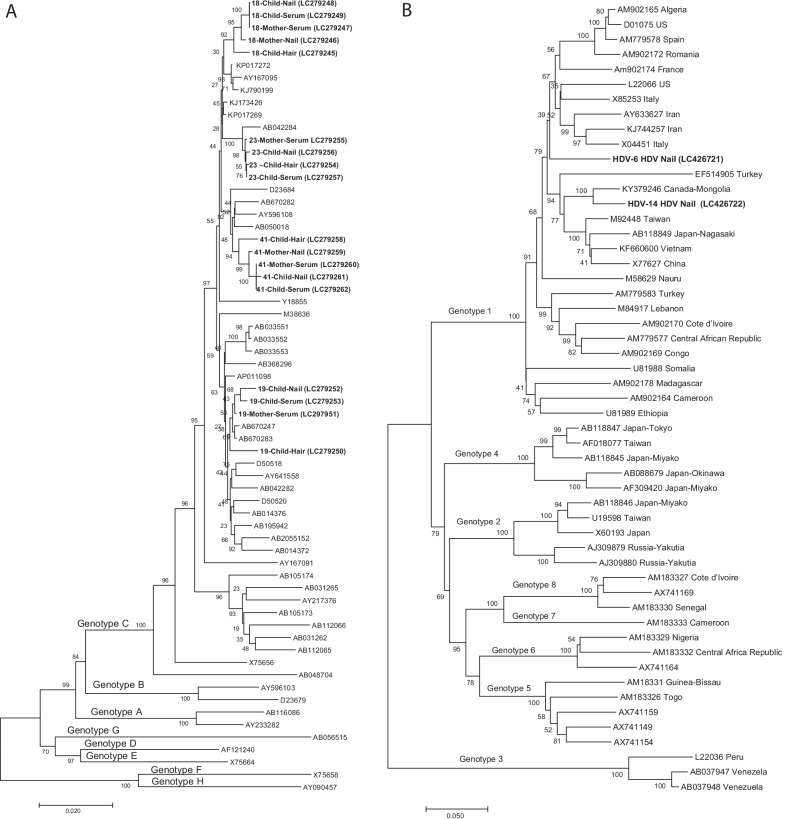
Phylogenetic analysis. **A** Phylogenetic analysis of mother-to-child transmission. The percentage of replicate trees in which the associated taxa clustered together in the bootstrap test (1000 replicates) is shown next to the branches. The tree is drawn to scale, with branch lengths in the same units as those of the evolutionary distances used to infer the phylogenetic tree. The analysis involved 64 nucleotide sequences. **B** Phylogenetic analysis of the HDV genome sequence. The percentage of replicate trees in which the associated taxa clustered together in the bootstrap test (1000 replicates) is shown next to the branches. The tree is drawn to scale, with branch lengths in the same units as those of the evolutionary distances used to infer the phylogenetic tree. The analysis involved 53 nucleotide sequences

The results of our phylogenetic tree analysis of the 18 complete genome sequences of HBV isolated from serum, nail, and hair HBV DNA are shown in Fig. 3A. All 18 of the sequences belonged to HBV genotype C. Four clusters were observed in the phylogenetic tree, and each cluster was made of the same-number child and mother. These findings indicate that a phylogenetic tree analysis using HBV DNA isolated from nails and hair can confirm the mother-to-child transmission of HBV.

The phylogenetic tree analysis results of the two complete genome sequences of HDV are shown in Fig. 3B. The two complete sequences belonged to genotype 1, which is predominant in Mongolia [32, 33]. The complete sequence from patient no. HDV-14 showed high similarity (94%) with that from a Mongolian individual living in Canada [34]. Although the patients' blood samples were not available, these findings suggest that nails can also be useful to identify the source of HDV infection.

### Indirect immunogold labeling EM results

**Fig. 4:**
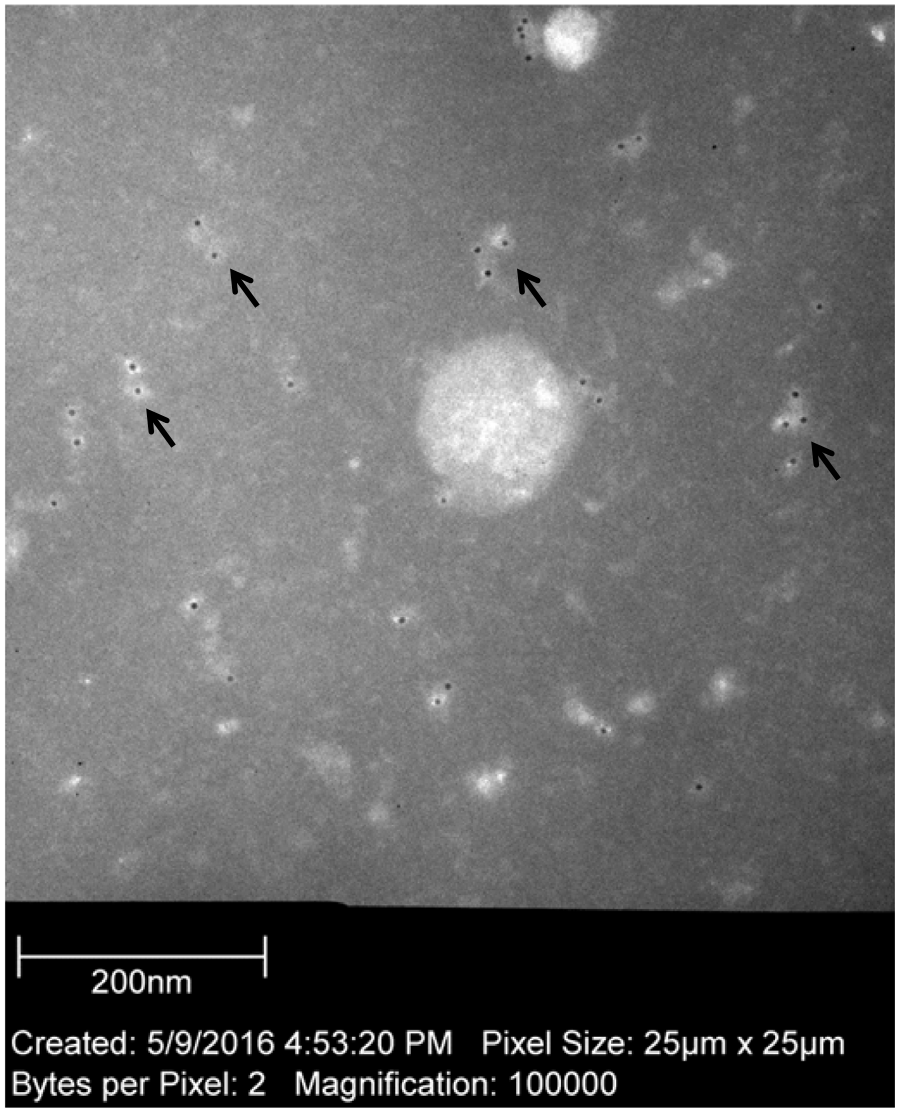
Indirect immunogold labeling electron microscopy**.** The arrows indicate the small round particles, which were approx. 20 nm in dia. and overlapped with 6-nm gold beads. Dane particles (42 nm dia.) are not present in this figure

We performed indirect immunogold labeling EM for the cell dissociation reagent, which was incubated with nail (HBV DNA level: 5.8 log copies/mL, HBsAg: 1.04 IU/mL) at 37 °C for 24 h. Many small round viral particles were observed to be attached to the secondary antibodies conjugated with 6 nm gold-bead in Fig. 4. The small round particles were approx. 20 nm in dia. However, Dane particles, which are mature virions and 42 nm in dia., were not observed by EM.

### Immunohistochemical staining of nail

**Fig. 5:**
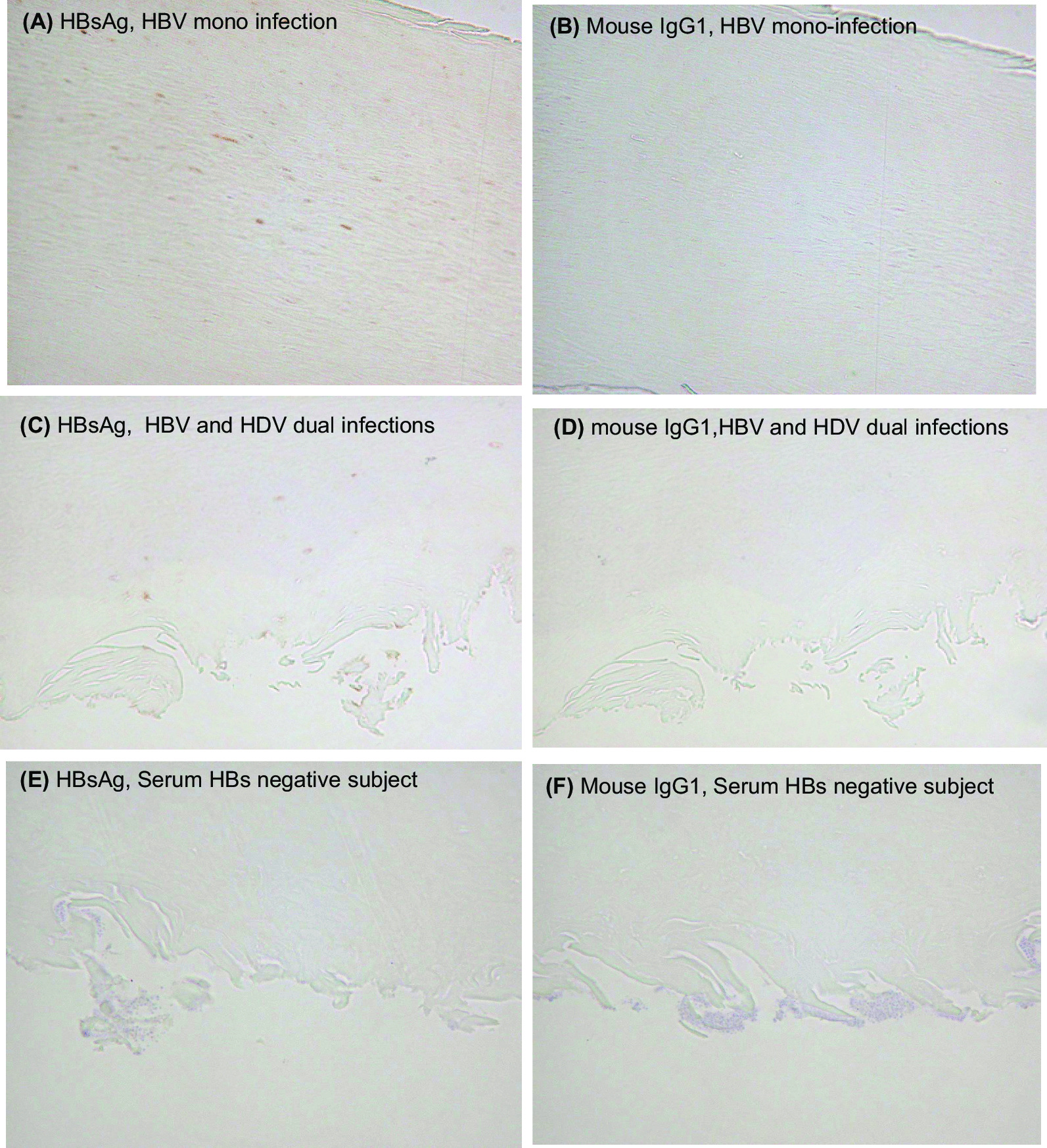
**A**, **B** Immunohistochemical staining of nail from a patient with chronic HBV mono-infection. Original magnification =  ×200. **A** HBsAg is stained brown. **B** The negative controls (mouse IgG1). **C**, **D** Immunohistochemical staining of fingernail from a patient with chronic HBV and HDV dual infections (no. HDV-14). Original magnification =  ×200. **C** HBsAg is stained brown. **D** Negative control (mouse IgG1). **E**, **F** Negative control. Immunohistochemical staining of fingernail from a serum HBsAg negative subject. Original magnification =  ×200

**Fig. 6:**
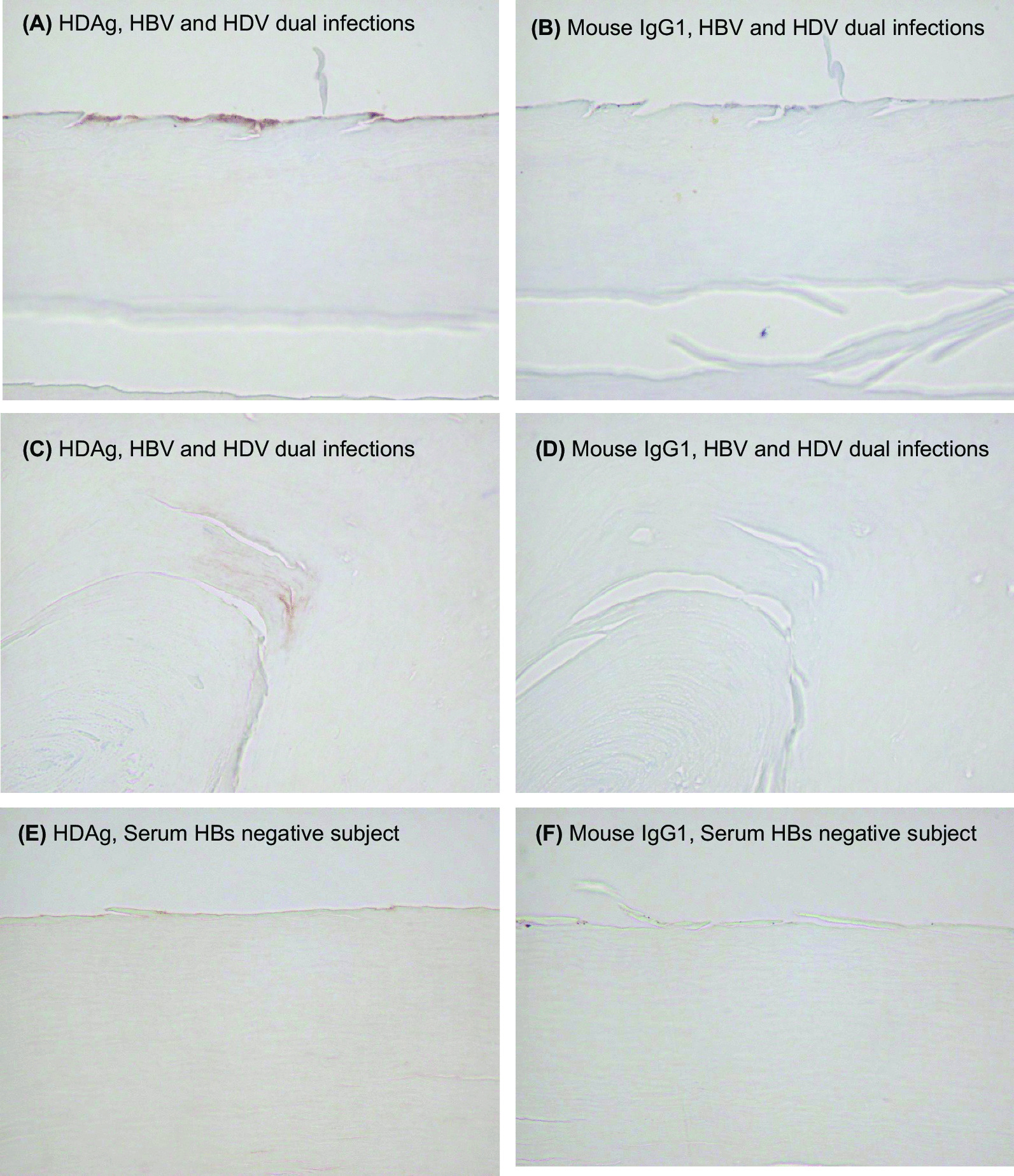
Immunohistochemical staining of fingernail from a patient with chronic HBV and HDV dual infections (no. HDV-14). Original magnification =  ×400. **A** Hepatitis D antigen was detected at the edge of the nail. **C** Hepatitis D antigen was detected inside of the nail. **B**, **D** are the negative controls (mouse IgG1). **E**, **F** Negative control. immunohistochemical staining of fingernail from a serum HBsAg negative subject. Original magnification =  ×400

The immunohistochemical staining of the nail samples (HBsAg level in nail: 1.33 IU/mL) from an HBV carrier is shown in Fig. 5. HBsAg stained brown was observed in the nail (Fig. 5A). In contrast, HBsAg was not observed in the nail using negative controls (Fig. 5B, D–F). These findings demonstrate that HBsAg is integrated into nail tissue. However, neither HBcAg nor SLC10A1 was detectable in the nail samples. We also performed immunohistochemical staining using nails from the patient with chronic HBV/HDV infection (no. D-14, HBsAg level in nail: 0.03 IU/mL). HBsAg stained brown was observed in the nail sample from the patient with chronic HBV/HDV infection (Fig. 5C). Moreover, HD antigen stained brown was also observed in the nail from the patient (Fig. 6A, C). HD antigen was not observed in the nail using negative controls (Fig. 6B, D–F).

### Inoculation of chimeric mice and real-time PCR

Because it was not deniable that the cell dissociating reagent was harmful for the chimeric mice, PBS incubated with nail was chosen for the inoculation. Two chimeric mice were intravenously inoculated once with 200 µL of filter-sterilized PBS, which was incubated with nail for 7 days (Table 4, Patient D). The PBS was positive for both HBV DNA and HBsAg (HBV DNA level: 4.8 log copies/mL, HBsAg: 0.1 IU/mL). Serum samples were taken from the three chimeric mice 1×/week for 10 weeks after the inoculation. A final total of 10 serum samples were taken from each mouse and examined by real-time PCR. There were no unexpected adverse events. None of the serum samples became positive for HBV DNA. Moreover, HBV DNA was not detectable in the liver tissue of the three chimeric mice. Although a positive control was not used for this experiment, these findings suggest that the nails of HBV carriers are not infectious material despite the presence of HBV DNA and HBsAg.

## Discussion

The present study demonstrated that HBV DNA was present in approx. 90% of the nails from HBV carriers. Moreover, HBV DNA was detected in approx. 80% of the hair samples from HBV carriers. These findings suggest that HBV DNA is integrated into hair as well as nails in the vast majority of patients with chronic HBV infection. A recent study reported that serum HBV DNA level > 10^7^ IU/mL was associated with 71.5% probability of fingernails being positive [19]. The detection rate of HBV DNA was a little higher in this study than that in the recent study. In this study, β-actin DNA was used for internal control and the DNA extraction kit specialized for nails and hair was used. Moreover, the median of serum HBV DNA level in the patients was > 9.0 log copies/mL. These factors could contribute the higher detection rate in this study. In addition to this study, our recent study showed that HBV DNA was detectable in nails and hair of children with acute HBV infection [26]. These findings strongly indicate that HBV DNA can be present in nails and hair.

HBV DNA was not detectable in the nails or hair from the patients who had low levels of HBV DNA in serum, whereas a high viral load in serum was significantly associated with the positivity of HBV DNA in both nails and hair. However, the HBV DNA level in serum was not correlated with that in nails or hair. This is because unlike blood, nails and hair have an upper limitation of HBV DNA. On the other hand, there was a significant association in HBV DNA level between the nails and hair. Both nails and hair are skin appendages that share with the skin a common origin from the ectodermal layer. Although the morphology of nail and that of hair differ, the biogenesis of nails and hair has many similarities [17, 35]. Therefore, a significant association could be observed in HBV DNA level between the nails and hair.

We observed that despite the rapid reduction of the serum HBV DNA level after the initiation of antiviral treatment, the HBV DNA level of nails and hair remained the same or gradually decreased. The HBV DNA level in nails and hair could reflect the exposure of matrix cells in nails and hair to HBV for a past time frame, like trace elements. The growth rate of hair is 6–15 mm per month, and the growth rate of fingernails is 3 mm per month [35, 36]. On the basis of the growth rate, nail clippings could retain HBV DNA at least several months after the disappearance of serum HBV DNA. Previously, we reported a mother-to-child transmission case in which a past time frame of nails contributed to the detection of vaccine scape mutant [37]. In the report, a 2-year-old boy was chronically infected through breakthrough infection and G145R vaccine escape mutant was detected in his serum. An HBV carrier mother treated with tenofovir after delivery. Although we tried to analyze HBV DNA from her serum, she became negative for serum HBV DNA due to antiviral treatment. However, HBV DNA was detectable in her nails and G145A vaccine escape mutant was identified in her nails. The past time frame is an advantage of nails and hair in clinical settings. Moreover, sampling of nails and hair is non-invasive and easy. These advantages are expected to contribute to basic research and clinical investigation.

The detection rate of HBV DNA in nails was higher than that in hair. Additionally, the HBV DNA levels in nails were significantly higher than that in hair. These findings suggest that nails are a more reliable material to extract DNA than hair. There are several possible explanations why these findings were observed. First, the quality of DNA varies in hair among the same individual’s samples [17]. The root of a hair, which is a metabolically activated site, contains high-quality DNA. However, the growth of hair is comprised of three phases called the anagen phase (the growth stage, lasting 3–7 years), the catagen phase (the degeneration stage, lasting for a few days to 2 weeks), and the telogen phase, which is a resting stage of variable periods [35, 38]. The difference in hair-follicle cycling could produce the variations of DNA quality among same individual's samples. Moreover, it is sometimes difficult to identify the root of a hair for DNA extraction. These variations could contribute to the level of HBV DNA in hair.

Secondly, hair is often exposed to chemicals such as hair treatment and hair dyes in daily life. These chemicals could influence the DNA extraction and PCR [17]. Thirdly, melanin derived from hair could be an inhibitor of a PCR [17, 39]. The density of melanocytes is higher in the hair bulb than the skin epidermis, and almost all Japanese individuals have black hair [40]. The concentration of melanin is higher in black hair than that in hair of other colors [41]. The melanin in black hair might reduce the efficiency of a PCR. Fourthly, the turnover of matrix cells, which form germinative tissue, from the birth to fully keratinized (death) in hair is 2.5 days, whereas the turnover of matrix cells in skin is up to 10 days [17]. The difference in the duration between hair’s ‘birth’ and ‘death’ might influence the exposure of matrix cells to HBV and the level of HBVDNA in hair.

In addition to the detection of HBV DNA in nails and hair, it is also important to determine whether HDV RNA is detectable in nails. If viral RNA could be detected, this technique could be applied to detect various DNA and RNA viral infections in nails in animals as well as humans. To clarify whether the viral RNA genome can be detected in nails, we examined nails from patients with chronic HBV/HDV infection. The positive rate of nail HBV DNA in the patients with chronic HBV/HDV infection was lower than that in the patients with chronic HBV mono-infection. It is likely that the low positive rate of nail HBV DNA in the patients with chronic HBV/HDV infection was due to their low levels of serum HBV DNA. Although the viral replication of HBV and HDV varies during the clinical course [42], the viral replication of HBV tends to be suppressed by HDV [43]. It is thus reasonable that the detection of HBV DNA in nails was not frequent in our patients with chronic HBV/HDV infection. Because the viral load of HDV in blood was relatively high in this group, before the experiment we expected that the positive rate of nail HDV RNA would be high. HDV genome was successfully detected in the nails, but the positive rate of nail HDV RNA was low. It is difficult to explain why nail HDV RNA was not frequently detected despite the high viral load in blood. Two possibilities may explain this finding. One is the fragile nature of RNA. In general, RNA is less stable than DNA. This fragility might reduce the positive rate of viral RNA in nails. The other possible reason is the method of RNA extraction from nails. Because nails are highly keratinized, the method of RNA extraction from nails has not yet been standardized. The method of RNA extraction used in this study might be insufficient to detect viral RNA.

The replication of HBV in the skin remains controversial. In 1985, Rosen et al. reported that HBsAg was detected by immunochemical staining in the epidermis of skin from a patient with acute HBV infection; however, HBcAg was not detected [10]. In 1993, Mason et al. reported that HBV RNA was detected by means of in situ hybridization in the epidermis of skin from a patient with chronic HBV infection. Although they reported that HBV-related skin lesions were associated with local viral replication, neither HBsAg nor HBcAg was detected in the patient’s skin by immunochemical staining [8]. Although the values of nail HBsAg were low in the present investigation, HBsAg was detectable in 59% and 53% of the nail samples from the patients with chronic HBV mono-infection and HBV/HDV dual infections, respectively. The existence of HBsAg and HD antigen in the nails was confirmed by immunochemical staining. HBcAg is a marker of active viral replication, but was not detected by the immunochemical staining of nails.

As a matter of public health, it is important to investigate whether nails are an infectious agent. We conducted an animal experiment to clarify the infectivity of nails from HBV carriers. In our previous study, we used isolated primary human hepatocytes from chimeric mice with humanized liver to investigate the infectivity of HBV [25]. The previous study suggested that high HBV DNA level (6 log copies/mL or more) was needed to infect isolated primary human hepatocytes from chimeric mice. However, the median HBV DNA level was 5 log copies/mL. Therefore, we used chimeric mice for the investigation of infectivity of HBV from nails. Because chimeric mice with humanized livers have been established as an animal model of HBV infection [44, 45], no positive control was used to save animal and laboratory costs. Because we suspected that the cell dissociation reagent could have harmful effects on the chimeric mice, we used PBS incubated with nail samples that contained both HBV DNA (4.8 log copies/mL) and HBsAg (0.1 IU/mL) for the inoculation. According to the results of a previous study, 10–100 copies were sufficient for the development of HBV infection in a chimeric mouse [45]. In this study, the mice were injected with approximately 10^4^ copies of HBV DNA. Because serum HBV DNA had been undetectable in the chimeric mice for 10 weeks after the inoculation, we concluded that nails from HBV carriers are not infectious agents. However, HBV cccDNA and HBV pgRNA was detected in one patient who showed the highest value of nail HBV DNA in this study. Because cccDNA is the template for the transcription of all viral mRNAs, the presence of the cccDNA might be associated with the highest values of nail HBV DNA in this study. Further investigations are required to determine whether HBV can replicate in nails.

Our study has several limitations. The mechanism of the viral entry of HBV into nails could not be clarified in this study. We hypothesized that NTCP was related to the entry of HBsAg into nail, although NTCP is expressed predominantly in liver tissue [5]. However, immunochemical staining failed to detect NTCP in the nails. Although mRNA was extracted from the nails, the PCR assay could not detect the complementary DNA coding for NTCP. In addition, although the growth rate of nails and hair may vary depending on age, gender and race, we did not evaluate the differences in these factors due to the small number of subjects in this study. It was reported that toenail (growth rate: 1 mm/month) is more reliable for the measurement of trace elements and less likely to be contaminated [14]. Although inverse PCR was performed to find virus-cell integration site in nails [46], we failed to detect integrated HBV DNA in the nails. We have tried to perform the in-situ hybridization for nails to prove the existence of HBV DNA in the nails. However, nails were always broken due to high temperature of in-situ hybridization. Moreover, sufficient significant data, which clarifies the mechanism of HBV in nails and hair, was not observed in this study. To obtain more significant results, a larger number of subjects and the improvement of investigation methods are necessary.

## Conclusions

Fingernails and hair are reservoirs of HBV DNA in patients with chronic HBV infection. HDV RNA was also detected in fingernails from patients with chronic HBV/HDV dual infections. Moreover, HBsAg was detectable in nails.

## Supplementary Information

**Additional file 1: Figure S1.** Real-time PCR for the detection of cccDNA and HBV RNA. **A** cccDNA was detected in the nail DNA treated with T5 exonuclease and quantified by real time PCR. The recombinant plasmid controls (copies/mL) were used for the quantification of cccDNA. **B** Real-time PCR for the detection of HBV RNA in serum. To confirm the specificity of reverse transcription and real-time PCR, HBV RNA was reverse transcribed with random sequence anchored HBV primer and random hexamers. Random hexamer was used as a negative control. HBV specific sequence was used for the forward primer. Random sequence was used for the reverse primer. Serum HBV RNA was detected in cDNA which was generated with random sequence anchored HBV primer, but not in cDNA which was generated with random hexamer. **C** Real-time PCR for the detection of HBV RNA in nails. Nail HBV RNA was detected in cDNA which was generated with random sequence anchored HBV primer, but not in cDNA which was generated with random hexamer. **D** The quantification of HBV RNA in nails. Transcripts were used as controls for the quantification of HBV RNA (copies/mL).

**Additional file 2: Table S1.** Primer sequences of nested PCR for hepatitis delta virus complete genome. The nucleotide positions of the primer sequences were based on the HDV complete genome (GenBank Accession No. KF660600).

## Data Availability

The datasets generated and/or analyzed during the current study are available in the DDBJ/EMBL/GenBank nucleotide sequence databases with the accession numbers LC279245-62 and LC426721-2.

## Abbreviations

cccDNA: Covalently closed circular DNA
EM: Electron microscopy
HBsAg: Hepatitis B surface antigen
HB pgRNA: Hepatitis B virus pregenomic RNA
HBV: Hepatitis B virus
HDV: Hepatitis delta virus
NTCP: Sodium taurocholate co-transporting polypeptide
PEG-IFN: Pegylated-interferon
