# Supplementary figures and images for: Signature of chronic hepatitis B virus infection in nails and hair
Source: BMC Infect Dis. 2022 May 4;22:431. doi: 10.1186/s12879-022-07400-8 (PMC9066816; doi:10.1186/s12879-022-07400-8)

## Slide 1
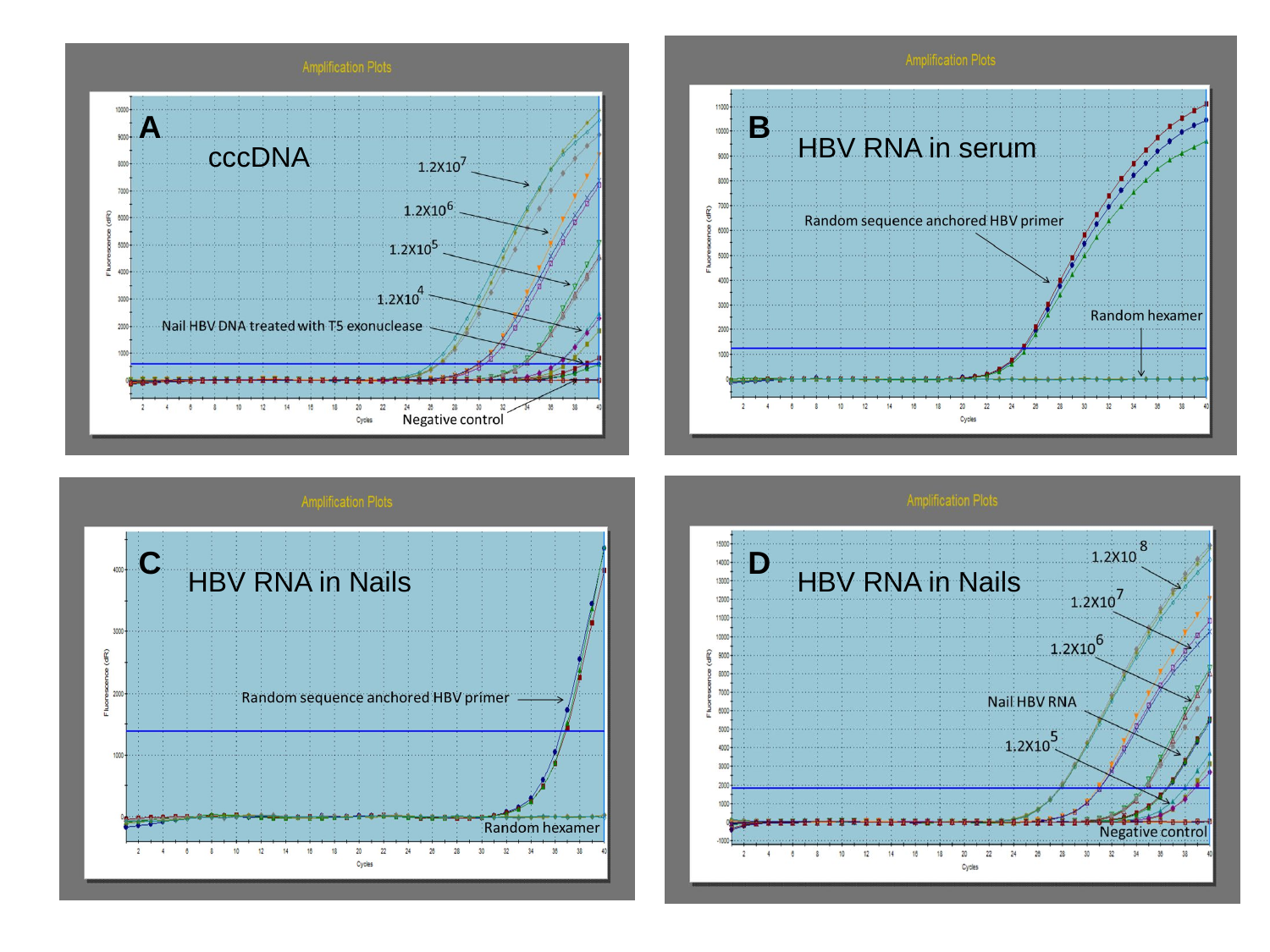

A
B
HBV RNA in serum
cccDNA
C
D
HBV RNA in Nails
HBV RNA in Nails

Supplement: Supplementary file 1 — Additional file 1: Figure S1. Real-time PCR for the detection of cccDNA and HBV RNA. A cccDNA was detected in the nail DNA treated with T5 exonuclease and quantified by real time PCR. The recombinant plasmid controls (copies/mL) were used for the quantification of cccDNA. B Real-time PCR for the detection of HBV RNA in serum. To confirm the specificity of reverse transcription and real-time PCR, HBV RNA was reverse transcribed with random sequence anchored HBV primer and random hexamers. Random hexamer was used as a negative control. HBV specific sequence was used for the forward primer. Random sequence was used for the reverse primer. Serum HBV RNA was detected in cDNA which was generated with random sequence anchored HBV primer, but not in cDNA which was generated with random hexamer. C Real-time PCR for the detection of HBV RNA in nails. Nail HBV RNA was detected in cDNA which was generated with random sequence anchored HBV primer, but not in cDNA which was generated with random hexamer. D The quantification of HBV RNA in nails. Transcripts were used as controls for the quantification of HBV RNA (copies/mL). [file 12879_2022_7400_MOESM1_ESM.pptx]
