# Supplementary material for: Signature of chronic hepatitis B virus infection in nails and hair
Source: BMC Infect Dis. 2022 May 4;22:431. doi: 10.1186/s12879-022-07400-8 (PMC9066816; doi:10.1186/s12879-022-07400-8)
Supplement: Supplementary file 2 — Additional file 2: Table S1. Primer sequences of nested PCR for hepatitis delta virus complete genome. The nucleotide positions of the primer sequences were based on the HDV complete genome (GenBank Accession No. KF660600). [file 12879_2022_7400_MOESM2_ESM.docx]

| **Table S1.**  Primer sequences of PCR for hepatitis delta virus complete genome | | | | |  |  |
| --- | --- | --- | --- | --- | --- | --- |
| **Fragment name** | **Primer name** | **Sequence** | **Nucleotide position** | **Ref.** | | |
| P1 | P1-1st forward | GGATGCCCAGGTCGGACCG | 848–866 | 29 | | |
|  | P1-1st reverse | AAGAAGAGRAGCCGGCCCGY | 1151–1170 |  |  |  |
|  | P1-2nd forward | ATGCCATGCCGACCCGAAGA | 880–899 |  |  |  |
|  | P1-2nd reverse | GGGGAGCGCCCGGDGGCGG | 1096–1114 |  |  |  |
| P2 | P2-1st & 2nd forward | GGACCCCTTCAGCGAACAG | 307–325 | 29 | | |
|  | P2-1st reverse | CACTCGGATGGCTAAGGGAG | 664–683 |  |  |  |
|  | P2-2nd reverse | GGCCATCAGGTAAGAAAGGA | 811–830 |  |  |  |
| P3 | P3-1st forward | GATGCTCCTCCCGATGTC | 576–593 | Present study | | |
|  | P3-1st reverse | TTTTCTCCCCAGAGTTGTCG | 958–977 |  |  |  |
|  | P3-2nd forward | TCCCGATGTCCGTCCAAC | 581–601 |  |  |  |
|  | P3-2nd reverse | TGAATAAAGCGGGTTTCCAC | 931–950 |  |  |  |
| P4 | P4-1st forward | CTATGGGAATCCCCGGTCT | 1005–1023 | Present study | | |
|  | P4-1st reverse | GGAGTCCCGGAAGGTTAAGA | 1478–1497 |  |  |  |
|  | P4-2nd forward | GTCCTGGTGAAGGGGGACT | 1048–1066 |  |  |  |
|  | P4-2nd reverse | GCTGGGAAACATCAAAGGAA | 1424–1443 |  |  |  |
| P5 | P5-1st & 2nd forward | TACCTCCATCTGGTCCGTTC | 1350–1369 | Present study | | |
|  | P5-1st reverse | CGTCCGTCTCTTGCTTTCTC | 111–130 |  |  |  |
|  | P5-2nd reverse | TTACCGTTTTCCCCTCTCG | 48–66 |  |  |  |
| P6 | P6-1st & 2nd forward | AGAGGAGACTGCTGGACTCG | 1639–1658 | Present study | | |
|  | P6-1st reverse | GCTTTCTTTGCTTTCCTCCTC | 358–378 |  |  |  |
|  | P6-2nd reverse | TCCTAGCATCTCTCCCTATCG | 397–417 |  |  |  |
|  | | | | | |  |
